# An Activity Index for Raw Accelerometry Data and Its Comparison with Other Activity Metrics

**DOI:** 10.1371/journal.pone.0160644

**Published:** 2016-08-11

**Authors:** Jiawei Bai, Chongzhi Di, Luo Xiao, Kelly R. Evenson, Andrea Z. LaCroix, Ciprian M. Crainiceanu, David M. Buchner

**Affiliations:** 1 Department of Biostatistics, Johns Hopkins University, Baltimore, Maryland, United States of America; 2 Division of Public Health Sciences, Fred Hutchinson Cancer Research Center, Seattle, Washington, United States of America; 3 Department of Statistics, North Carolina State University at Raleigh, Raleigh, North Carolina, United States of America; 4 Department of Epidemiology, Gillings School of Global Public Health, University of North Carolina–Chapel Hill, Chapel Hill, North Carolina, United States of America; 5 Division of Epidemiology, Department of Family Medicine and Public Health, University of California San Diego, La Jolla, California, United States of America; 6 Department of Kinesiology and Community Health, University of Illinois at Urbana-Champaign, Champaign, Illinois, United States of America; Indiana University, UNITED STATES

## Abstract

Accelerometers have been widely deployed in public health studies in recent years. While they collect high-resolution acceleration signals (e.g., 10–100 Hz), research has mainly focused on summarized metrics provided by accelerometers manufactures, such as the activity count (AC) by ActiGraph or Actical. Such measures do not have a publicly available formula, lack a straightforward interpretation, and can vary by software implementation or hardware type. To address these problems, we propose the physical activity index (AI), a new metric for summarizing raw tri-axial accelerometry data. We compared this metric with the AC and another recently proposed metric for raw data, Euclidean Norm Minus One (ENMO), against energy expenditure. The comparison was conducted using data from the Objective Physical Activity and Cardiovascular Health Study, in which 194 women 60–91 years performed 9 lifestyle activities in the laboratory, wearing a tri-axial accelerometer (ActiGraph GT3X+) on the hip set to 30 Hz and an Oxycon portable calorimeter, to record both tri-axial acceleration time series (converted into AI, AC, and ENMO) and oxygen uptake during each activity (converted into metabolic equivalents (METs)) at the same time. Receiver operating characteristic analyses indicated that both AI and ENMO were more sensitive to moderate and vigorous physical activities than AC, while AI was more sensitive to sedentary and light activities than ENMO. AI had the highest coefficients of determination for METs (0.72) and was a better classifier of physical activity intensity than both AC (for all intensity levels) and ENMO (for sedentary and light intensity). The proposed AI provides a novel and transparent way to summarize densely sampled raw accelerometry data, and may serve as an alternative to AC. The AI’s largely improved sensitivity on sedentary and light activities over AC and ENMO further demonstrate its advantage in studies with older adults.

## Introduction

**Fig 1:**
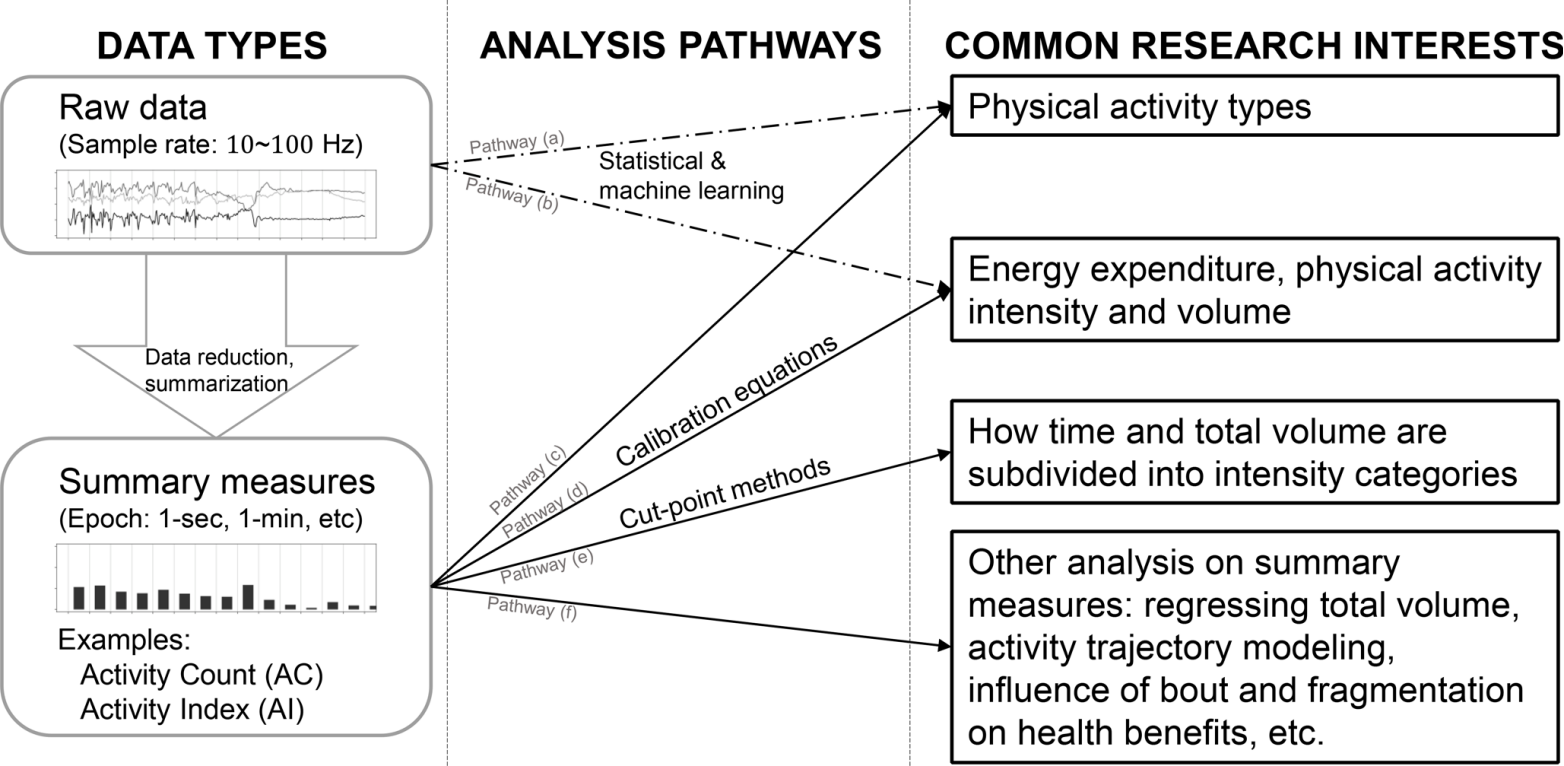
A general framework for accelerometer-related studies. The left panel illustrates two general data types: raw data and summary measures. The right panel shows 4 common research interests. The mid panel contains 6 common analysis pathways between the data and the research interests.

Accelerometers are now commonly used to measure physical activity, and are embedded both in research and commercial devices [1–6]. Fig 1 provides a conceptual analytic framework for accelerometer data in physical activity studies. While most modern accelerometers collect high-resolution signals (e.g., 10–100 Hz), the most commonly used data output consists of summary measures over user-defined epochs (e.g., 1 minute). These measures are obtained by processing raw data using software developed by device manufacturers (see the panel “DATA TYPES” in Fig 1). For example, both ActiGraph GT3X+ (ActiGraph, Pensacola, FL) and Actical (Phillips Respironics, Bend, OR) software use proprietary algorithms to calculate an “activity count” (AC)[2,7], but the two AC are not equivalent. Thus, AC has become an umbrella term for a large number of proprietary algorithms, which leads to widespread confusion among health researchers. Summary measures, such as AC, have been widely used either directly as a measure of physical activity volume or intensity, or indirectly as a predictor of energy expenditure (see analysis pathways (c), (d), (e), (f) in Fig 1) [8–13].

The main reason for using such summary measures is that traditionally they were the only output of research-grade accelerometers. An important area of research is concerned with establishing the connection between summary measures of accelerometry and standard measures of physical activity intensity (metabolic equivalents (MET)) and physical activity volume (MET-min) (see review [14]). One goal of that research is to translate accelerometry summaries into physical activity intensity categories: sedentary, light, moderate, and vigorous activity. Thus, there are many calibration studies designed to identify the cut-points for physical activity counts that correspond to intensity categories [12,15–19], as well as studies designed to translate count data into METs and caloric expenditure [1,20,21].

More recently, high-resolution raw accelerometry data has become available on various devices, including on the ActiGraph GT3X+ and GENEActiv (Activinsights Ltd, Cambridgeshire, UK) accelerometers. Rather than relying on manufacturer software, researchers have started to develop new analytic approaches for the raw data. Of particular interest has been physical activity type recognition (pathway (a), [22–27]) and energy expenditure or MET prediction (pathway (b), [26,27]) using statistical and machine learning approaches. However, little research has been focused on developing an explicit, open-source, and reproducible summary metric based on raw data as an alternative to existing metrics (e.g., AC). The need for such measures is central to physical activity research, as current definitions of AC are proprietary and device- and software-specific [7,28]. A transparent and publicly available summary metric derived from raw data has the potential to allow comparisons of results across studies that use different accelerometers, improve translation among studies, and allow a more uniform interpretation of results.

Two notable summary metrics based on raw accelerometry data are Activity Intensity (AI_0_) by Bai et al. [28] and Euclidean Norm Minus One (ENMO) by van Hees et al. [29]. The AI_0_ measures the amplitude of the raw accelerometry signal relative to its amplitude distribution at rest, while ENMO is the vector magnitude of raw signals after removing 1*g* (one Earth standard gravitational unit). Both AI_0_ and ENMO are designed to quantify the magnitude of acceleration during a given epoch. AI_0_ has a publicly available formula and clear interpretation. However, its reliance on the choice of inactive periods and a threshold for systematic noise make it relatively difficult to implement in large studies. ENMO was also reported to be highly associated with physical activity energy expenditure [29,30], but it was not directly compared with AC. In this paper, we propose a new physical activity index (AI), which substantially improves AI_0_ by reducing its reliance on identifying all rest periods, making it rotationally invariant, and ensuring the consistency of definition across time domains. We show that AI outperforms both AC and ENMO in terms of prediction of physical activity energy expenditure and classification of physical activity intensity.

## Materials and Methods

### Participants

The Objective Physical Activity and Cardiovascular Health (OPACH) Study is an ancillary study of the Women’s Health Initiative 2010−2015 Long Life Study. The OPACH included a calibration sub-study, where 200 women aged 60 to 91 years old were invited to participate in one laboratory session to calibrate accelerometry counts to energy expenditure. This sub-study was approved by the Institutional Review Boards from each data collection site and by the Women’s Health Initiative Clinical Coordinating Center. Participants were asked to visit the study clinic site where they signed an informed consent form and completed a brief questionnaire.

### Accelerometry

ActiGraph GT3X+, a tri-axial accelerometer, was used in OPACH to measure physical activity. It was set to collect 30 Hz raw acceleration time series (*x*, *y* and *z* axes). GT3X+ features an “idle sleep mode” [31], which means when an internal algorithm detects no movement for 10 consecutive seconds, the last sampled raw acceleration value during the 10^th^ second is repeated infinitely until movement is detected by the algorithm again. The ActiLife 6 companion software could then calculate axis-specific AC (*AC*_*x*_, *AC*_*y*_ and *AC*_*z*_ for 3 axes) and the AC Vector Magnitude ($\sqrt{{AC}_{x}^{2}+{AC}_{y}^{2}+{AC}_{z}^{2}}$, which is the square root of the sum of square of the axis-specific AC) using the raw acceleration time series. The AC Vector Magnitudes are referred to as AC in the rest of this manuscript. An optional data processing procedure called “Low Frequency Extension (LFE)” [32] was also implemented while calculating AC. This is a variation on the AC measurement designed to improve AC’s sensitivity to sedentary and light activities [33]. AC with and without LFE were both calculated and used in this paper.

### Data collection

The participants performed several standardized tasks while simultaneously wearing an accelerometer, a heart rate monitor, and a portable indirect calorimeter to measure oxygen uptake. The hip-worn accelerometer was placed at the iliac crest and secured with a belt. Oxygen uptake (VO_2_) and heart rate were measured continuously during the physical activity tasks using the Oxycon Mobile (CareFusion, Rolle, Switzerland), a portable, battery operated, breath-by-breath metabolic unit.

The tasks of the calibration study were selected to vary in intensity from sedentary to moderate intensity in older women. Women provided Borg ratings of perceived exertion (RPE) [34] for each task to ensure level of effort did not exceed moderate-intensity. With the exception of treadmill walking at different speeds, participants rested ≥ 2 minutes between activities so that heart rate could return to within 10 beats/minute of resting heart rate. Simultaneous measurements of accelerometer counts, heart rate, and VO_2_ were recorded during the entire period for each physical activity. The duration of tasks was chosen to achieve steady rate metabolism for measurement of task-specific oxygen uptake. The participants performed tasks in the following order: watching DVD while sitting quietly (alias: DVD), assembling puzzle while sitting (alias: PUZZ), washing dishes while standing (alias: DISH), doing laundry while standing (alias: LAUD), 400-meter walking (alias: WALK), dust mopping while standing (alias: MOP), treadmill walking at 1.5mph (alias: TM15), and treadmill walking at higher speed, either 2.0mph (alias: TM20) or 2.5mph (alias: TM25) depending on the RPE. Determination of a 2.0 mph vs. a 2.5 mph pace for the second walking stage was based on participant’s RPE after 5 minutes into the 1.5 mph walk. At this point, women reporting a RPE of ≤ 11 walked at the 2.5 mph pace, while those reporting a RPE of 12−14 walked at the 2.0 mph pace. Women with a RPE >14 did not continue with the faster paced treadmill walk.

In the rest of the paper, the physical activity types are referred to by their aliases. The raw accelerometry data were used to compute AI and ENMO. The VO_2_ was converted to average energy expenditure during each activity in METs, by dividing the oxygen intake by 3.5 mL/(kg∙min). In addition, standard measurements such as weight, height and blood pressure were taken during the laboratory visit. More details about these measurements and protocol can be found elsewhere [19].

### The new Activity Index

Raw accelerometer data measure total acceleration from both device movement and gravity; the latter of which is always 1*g* downward. As previously reported [28], the variability of raw acceleration signals (standard deviation or variance) in short epochs (e.g., 1 second) removes gravity and provides a summary measure of movement intensity. The standard deviation captures the magnitude of the signals’ oscillation. When the frequency of such oscillation increases (e.g., when the accelerometer wearer switched from walking to running), the standard deviation can detect the increased variability of the signals, while the mean may not change accordingly. Thus, we chose to use the variance of raw accelerometry data along the three axes as building blocks to construct the proposed metric.

Specifically, let $\sigma_{im}^{2}(t;H)$ denote the variance of participant *i*’s acceleration signals along axis *m* (*m* = 1,2,3) in the window of length *H* starting at *t*. We then aggregate the variability of three axes by taking their sum, $\sigma_{i1}^{2}(t;H)+\sigma_{i2}^{2}(t;H)+\sigma_{i3}^{2}(t;H)$. The sum was normalized using the systematic noise variance denoted by ${\bar{\sigma }}_{i}^{2}=\sigma_{i1}^{2}+\sigma_{i2}^{2}+\sigma_{i3}^{2}$, so that it yields zero values when the device is not moving. ${\bar{\sigma }}_{i}^{2}$ depends on the accuracy of the device and can be calculated using raw data in periods while the accelerometer is not moving. Specifically, $\sigma_{im}^{2}$ (*m* = 1,2,3) is the average of $\left\{\sigma_{im}^{2}(t;H);t\in T_{i}\right\}$ where $T_{i}$ stands for collection of time points *t* when the accelerometer is not moving. The proposed Activity Index, $AI_{i}^{ABS}(t;H),$ for an epoch of length *H* starting at time *t* is defined by

$$AI_{i}^{ABS}(t;H)=\sqrt{\max\left(\frac{1}{3}\{\overset{3}{\underset{m=1}{\sum }}\sigma_{im}^{2}(t;H)-{\bar{\sigma }}_{i}^{2}\},0\right)}.$$ (1)

The AI captures the variability of device acceleration in excess of systematic noise and has the same unit as “g”.

In practice, we found that the AI is typically in a narrow range, as expressed in unit of “g”, especially for sedentary to light activities. To enhance interpretability, we also present a modified version of AI on a relative scale. Specifically, we further standardize the AI using the systematic variance ${\bar{\sigma }}_{i}^{2}$,

$$AI_{i}^{REL}(t;H)=\sqrt{\max\left(\frac{1}{3}\{\overset{3}{\underset{m=1}{\sum }}\frac{\sigma_{im}^{2}(t;H)-{\bar{\sigma }}_{i}^{2}}{{\bar{\sigma }}_{i}^{2}}\},0\right)},$$ (2)

so that an AI value of 1 is equivalent to the smallest amount of variability detectable by the device. The values of the relative scale AI spread in a wider range similar to AC, and might be preferred by some researchers. For studies that utilize the same accelerometry device for all participants, two versions of the AIs are directly proportional, with the constant of proportionality equal to ${\bar{\sigma }}_{i}^{2}$, so their performances are equivalent. In the application to the OPACH study, we reported results using the relative scale AI in the application for ease of presentation and interpretation. Detailed definitions of all these quantities are provided in the Supporting Information.

The newly proposed AI has three desirable properties: ease of implementation, additivity, and rotational invariance. As these properties hold for AI in both absolute and relative scale, we denote the new AI as $AI_{i}^{new}(t;H)$ regardless of its scale. With an explicit formula, $AI_{i}^{new}(t;H)$ could be implemented in a computationally efficient way for large epidemiology studies with tens of thousands of participants wearing accelerometers. For additivity, we defined the second-by-second $AI_{i}^{new}(t;H_{s})$ to be the finest level for computing AI, where *H*_*s*_ was the window size for one second. Any aggregated AI (e.g., 1-minute AI or $AI_{i}^{new}(t;60H_{s})$) was obtained by summing up all the adjacent 1-second AIs within that period (e.g., $AI_{i}^{new}(t;60H_{s})=\sum_{s=1}^{60}AI_{i}^{new}(t+s-1;H_{s})$). Rotational invariance means that the AI summarizes the magnitude of movement over three axes, regardless of whether the orientation of the device.

These properties are described in the discussion, while the technical details and proofs are included in the Supporting Information. Note that although previously proposed AI_0_ [28] was also based on standard deviation of acceleration signals, it had several drawbacks and did not possess the three properties discussed above. Specifically, AI_0_ requires a participant-specific tuning parameter for the metric normalization, while AI only requires a device- or study-specific tuning parameter. Unlike AI, AI_0_ does not guarantee rotational invariance because it combines variability from 3 axes using the sum of standard deviations instead of the sum of variances. More details on the difference between AI and AI_0_ is included in the Supporting Information.

### Statistical analysis

#### Data processing

Among 200 women from the OPACH calibration study, 194 had complete raw accelerometry data available, which were used in our analysis. Second-by-second AI was computed for each participant during each physical activity. Due to the “idle sleep mode” of the ActiGraph, the 10-second periods in the beginning of the selected non-wear (idle) periods were used to estimate ${\bar{\sigma }}_{i}$. We calculated ${\bar{\sigma }}_{i}$ for 10 participants and found them to be very close to each other. Such consistency of ${\bar{\sigma }}_{i}$ across different participants (or essentially, devices) allowed us to combine them into $\bar{\sigma }=\sum_{i=1}^{I}{\bar{\sigma }}_{i}/I$, which was a study-specific systematic variation. Using $\bar{\sigma }$, second-by-second $AI_{i}^{new}(t;H_{s})$ was computed for Participant *i* at time *t*. Second-by-second ENMO was computed by calculating the average of $\left\{\max\left[0,\sqrt{\sum_{m=1}^{3}{\left\{X_{m}(t+s-1)\right\}}^{2}}-1\right]|s=1,2,\ldots ,H_{s}\right\}$ during each one-second window [*t*,*t* + *H*_*s*_ − 1] [30], where *X*_1_(*t*), *X*_2_(*t*) and *X*_3_(*t*) are the raw acceleration signals of each axis and *H*_*s*_ is the window size for one second. The corresponding AC and AC with LFE at each second were computed using the ActiLife software.

#### Directly comparing AI, AC and ENMO

Second-by-second AI, AC, and ENMO measurements were compared using different approaches. First, scatterplots of AI versus AC, and AI versus ENMO, for randomly selected participants were explored. Second, for each of AI, AC, AC (LFE), and ENMO, a boxplot of pooled metrics across all participants was generated for all 9 physical activities. Third, receiver operating characteristic (ROC) analyses were conducted to assess and compare the performance of AI, AC and ENMO in distinguishing different activity types. More specifically, we illustrated comparisons with examples of 4 pairs of activities: DVD vs. DISH, DVD vs. LAUD, DVD vs. PUZZ and WALK vs. MOP. The area under the ROC curves (AUC) was used to evaluate the prediction performance of each measurement, as it represents the accuracy of the test to discriminate between two samples, with values significantly greater than 0.5 indicating better discrimination than by chance alone.

#### Comparing MET prediction performance of AI, AC and ENMO

We compared AI, AC, and ENMO in terms of their predictive performance of energy expenditure, as measured by a portable indirect calorimeter in METs. Median METs during each activity were analyzed together with median AI, AC, and ENMO during each activity type. Scatterplots of AI, AC, and ENMO versus METs were used for visual inspection of these associations, with Pearson correlation coefficients reported. We also evaluated the performance of AI, AC, and ENMO when differentiating between activities of different intensities as defined by thresholds on METs. Sedentary behaviors were defined as those with MET< 1.5, light activities as those with MET∈ [1.5,3) and moderate-to-vigorous activities as those with MET≥ 3. AUC was used to compare the prediction performance of these metrics to distinguish between activities performed at different levels of energy expenditure.

#### Software

AC and AC (LFE) were computed using ActiLife (version 6.11.8; ActiGraph, Pensacola, FL). AI and ENMO computation, as well as the statistical analysis were performed in R (version 2.15.3; R Foundation for Statistical Computing, Vienna, Austria). The R package for AI computation is available on GitHub (https://github.com/javybai/ActivityIndex).

## Results

### Summary statistics

The 194 women used in our analysis had a mean age of 75.4 years (standard deviation 7.7), with 21.6% (*n* = 42) between 60–69 years, 44.8% (*n* = 87) between 70–79 years, 31.4% (*n* = 61) between 80–89 years, and 2.1% (*n* = 4) between 90–91 years. For body mass index, the participants were evenly distributed across normal weight, overweight, and obesity categories (*n* = 68, 60 and 63, respectively), while 3 participants were underweight.

Summary statistics (mean and standard deviation) for AI, AC, AC (LFE), and ENMO from the study are shown in Table 1. For sedentary, light, and moderate activities, the mean of AI and both ACs increased in the order of the energy cost of the activities: DVD, PUZZ, DISH, LAUN and MOP. However, the mean ENMO for the first four activities were similar, suggesting that ENMO may underperform the other metrics in terms of distinguishing between types of sedentary and light activities. For the three treadmill walking speeds, all metrics performed as expected, increasing as the speed increased from 1.5mph to 2.5mph. However, the ratio of the mean divided by the standard deviations of ENMO and both ACs were substantially larger than that of AI for each activity, indicating smaller heterogeneity for AI.

**Table 1:** Summary Statistics of AI, AC, AC (LFE), and ENMO of each activity.

Activity Type	Number of Participants	AI (1 sec) Mean (SD)	AC (1 sec) Mean (SD)	AC (LFE*) (1 sec) Mean (SD)	ENMO* (1 sec) Mean (SD)
DVD*	194	0.61 (3.03)	0.42 (5.06)	0.53 (5.35)	0.001 (0.004)
PUZZ*	193	5.20 (5.69)	2.21 (9.20)	3.39 (10.04)	0.001 (0.004)
DISH*	194	9.33 (7.66)	4.53 (12.29)	6.43 (13.17)	0.002 (0.007)
LAUN*	194	13.53 (9.73)	12.15 (19.79)	15.38 (20.19)	0.002 (0.006)
MOP*	193	25.57 (13.55)	29.69 (25.22)	33.73 (24.66)	0.011 (0.015)
WALK*	190	61.88 (19.78)	43.49 (19.41)	48.06 (18.94)	0.073 (0.033)
TM15*	171	41.62 (10.37)	24.61 (16.15)	29.80 (15.41)	0.041 (0.018)
TM20*	53	49.30 (13.30)	31.02 (19.45)	36.18 (18.75)	0.052 (0.021)
TM25*	90	63.60 (15.08)	42.02 (15.59)	46.62 (15.19)	0.076 (0.025)

*Abbreviations: Activity count, AC; Activity Index, AI; 400-meter walking, WALK; assembling puzzle while sitting, PUZZ; doing laundry while standing, LAUD; dust mopping while standing, MOP; Euclidean Norm Minus One, ENMO; Low Frequency Extension, LFE; treadmill walking at 1.5mph, TM15; treadmill walking at 2.0mph, TM20; treadmill walking at 2.5mph, TM20; washing dishes while standing, DISH; watching DVD while sitting quietly, DVD.

### Directly comparing AI, AC and ENMO

**Fig 2:**
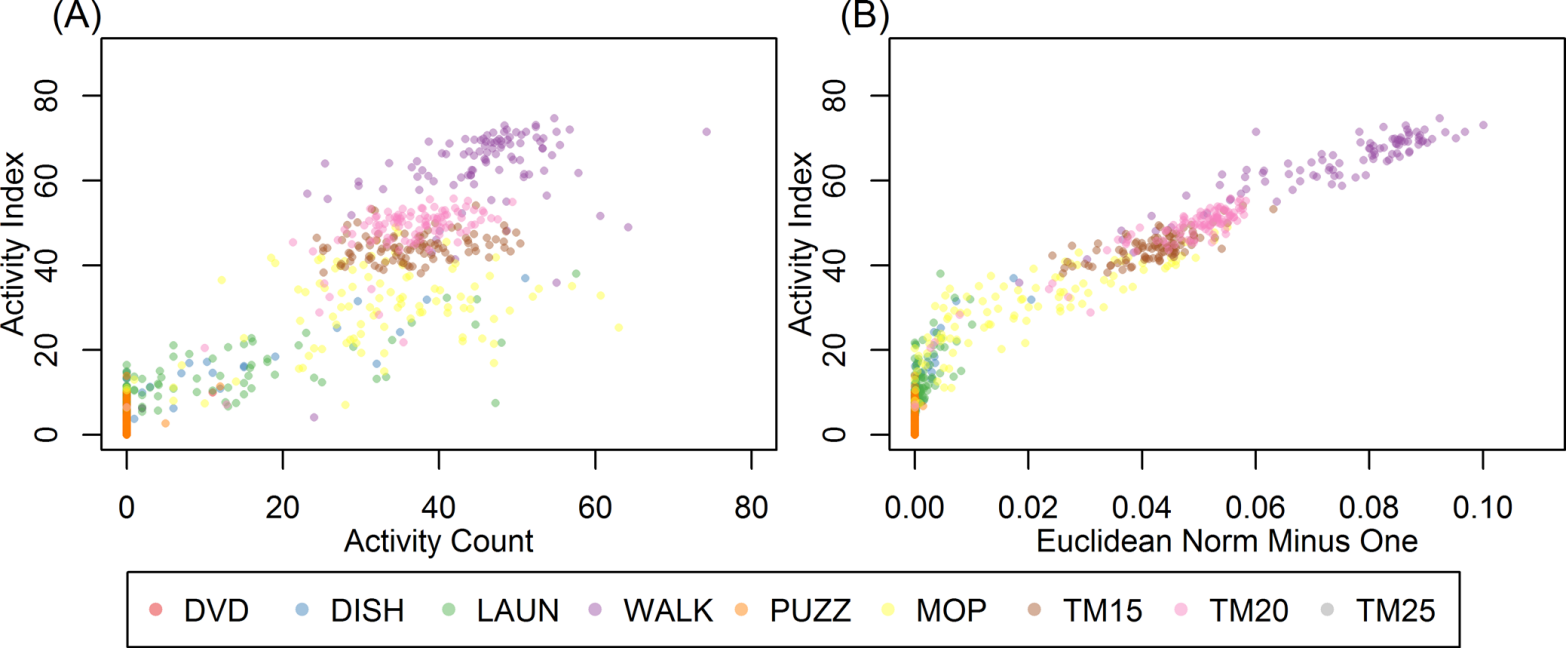
Scatterplots of Activity Index (AI, *y*-axis) versus activity count (AC, *x*-axis) (A) and AI (*y*-axis) versus Euclidean Norm Minus One (ENMO, *x*-axis) (B) for a randomly selected participant. Each point representing activity summery metrics in a 1-second interval. The points were rendered in different colors to represent different activity types. A random sample of 100 seconds were shown for each activity to reduce over-plotting.

Fig 2 displays second-by-second scatterplots of AI (*y*-axis) versus AC (*x*-axis) (Fig 2A) and AI (*y*-axis) versus ENMO (*x*-axis) (Fig 2B) for a randomly selected participant. The dots were rendered in different colors to distinguish among different activity types (one color per activity). To reduce over-plotting, we randomly sampled 100 seconds from each activity and only displayed the AI, AC and ENMO during these sampled seconds. The figure shows that ACs and ENMOs were often equal or very close to 0 for sedentary behaviors such as DVD and PUZZ. For light intensity activities including DISH and LAUN, AC displayed a wide spread in the range 0–60 with many zero values, while AI values were mostly nonzero and tended to be more clustered for each activity type. ENMO was highly correlated with AI for moderate activities (MOP, WALK, TM15, TM20 and TM25).

**Fig 3:**
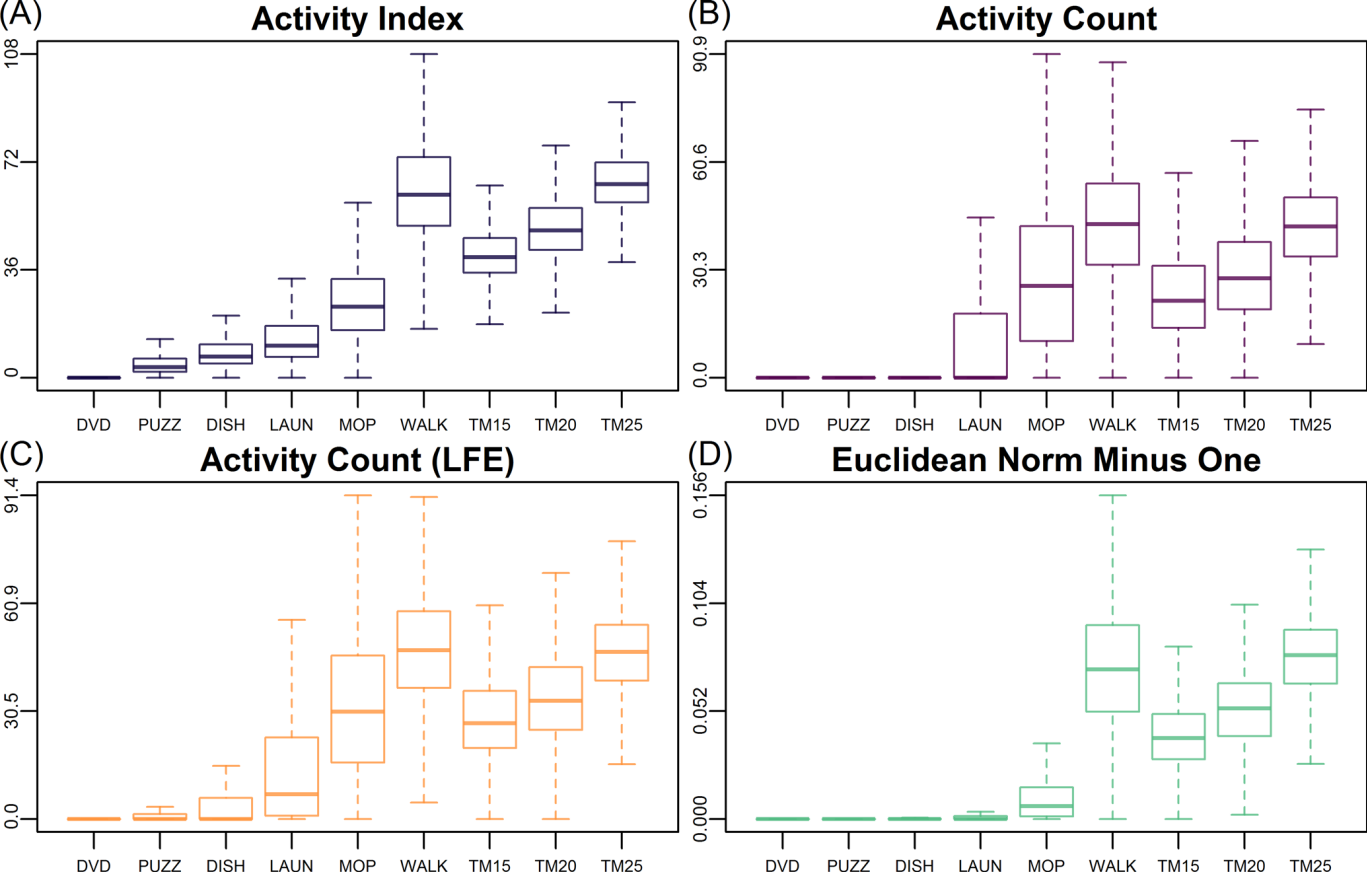
Comparison of the boxplots of Activity Index (AI), activity count (AC), AC with Low Frequency Extention (LFE) and Euclidean Norm Minus One (ENMO) during different types of activities. Outliers outside of the upper and lower whiskers are omitted. Each type of summary metric from all the participants were pooled together and plotted according to the type of activity.

Fig 3 illustrates the distribution of AI, AC, AC (LFE), and ENMO (after pooling all participants) for each activity. It confirmed that both AC and ENMO (Fig 3B and 3D) had values very close to 0 for sedentary and light activities (such as DVD, PUZZ, DISH, and LAUN). Though LFE increased AC’s sensitivity to sedentary and light activities (Fig 3C), there were still substantial zero counts for DVD, PUZZ, and DISH (with median close to zero). In contrast, AI in Fig 3A displayed distributions that were more separable for different activities, as the median AI values increased with activity intensity. For high light to moderate intensity physical activities, such as 400-meter walking and treadmill walking, the values of all four metrics were more concentrated and increased with gait speed. These observations implied that AI provides summary metrics for raw accelerometry signals that are more likely to be distinguishable among activities.

**Fig 4:**
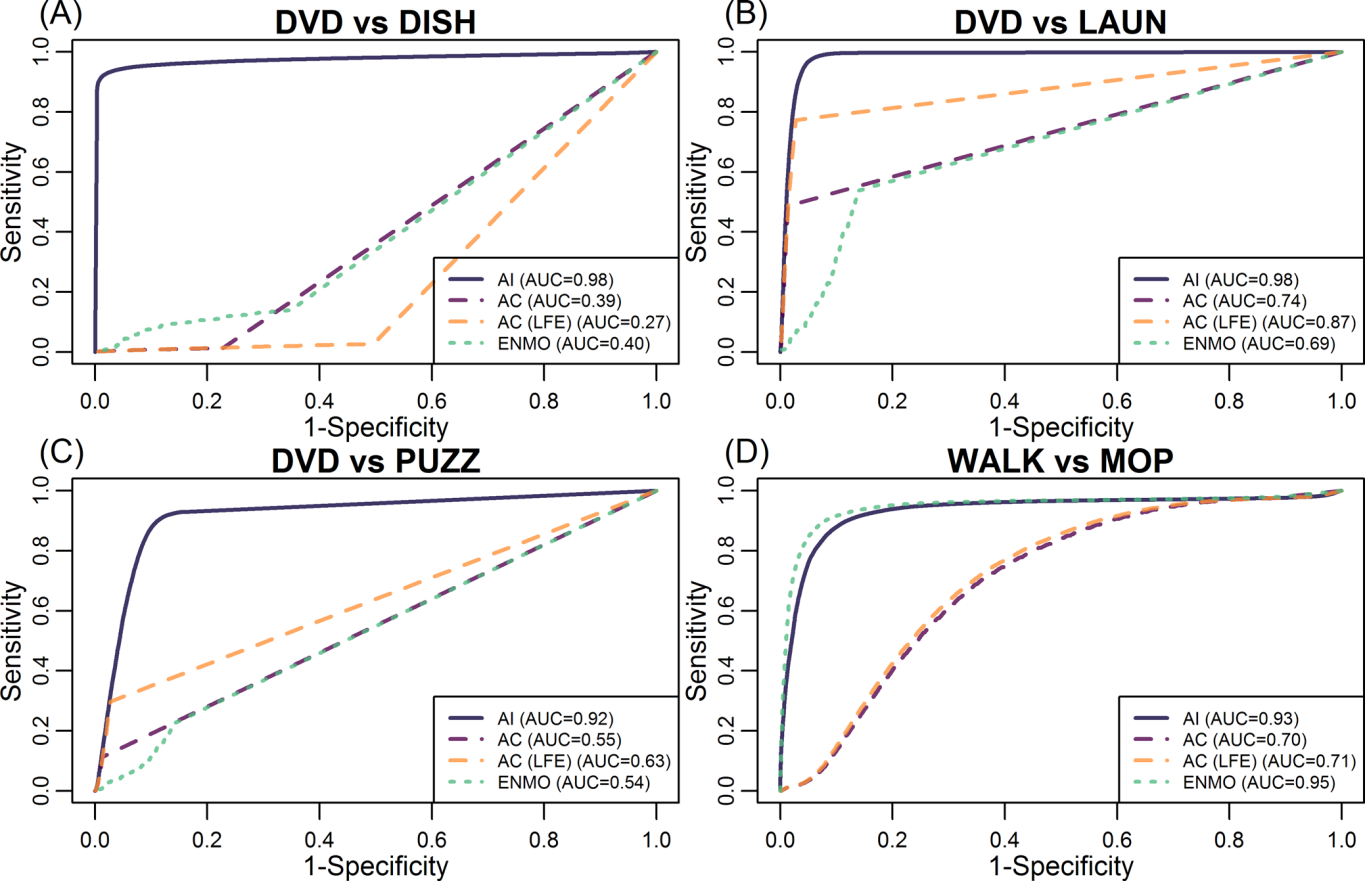
The “receiver operating characteristic” (ROC) curves for distinguishing four pairs of activity types, using Activity Index (AI, solid curves) or activity count (AC, dashed curves in different color for AC with and without Low Frequency Extension (LFE)) or Euclidean Norm Minus One (ENMO, dotted curves), respectively. The corresponding area under the curve (AUC) of each ROC curve is given in the legend section.

Fig 4 displays the four ROC plots of distinguishing various types of sedentary to light activities using AI, AC and ENMO. The solid, purple dashed, orange dashed, and dotted curves were ROC curves of AI, AC, AC (LFE), and ENMO, respectively. The dashed and dotted curves in Fig 4A and 4C were closer to the diagonal line (equivalent to a random guess classifier), while the corresponding solid curve was much higher overall. It indicated that neither AC nor ENMO could effectively differentiate DVD from DISH or PUZZ (AUC smaller than/close to 0.50), whereas AI performed much better with an AUC greater than 0.90. The predictive performance of AC and ENMO increased with METs (in the order of PUZZ, DISH and LAUD), but AI had substantially higher AUCs in all cases. In general, AC with LFE had greater AUC than both AC without LFE and ENMO, corresponding to better predictive performance for sedentary and light activities (Fig 4A, 4B and 4C). Fig 4D displays the performance of AI and AC for a pair of moderate to vigorous physical activities (MVPA), MOP versus WALK. For this pair of activities of the prediction performance of AC with and without LFE was very close (AUCs ~ 0.70–0.71). This suggests that LFE enhanced the prediction performance of AC for sedentary to light activities but not for moderate activities. AI and ENMO out-performed both versions of AC in this case, with an AUC of 0.93 and 0.95, respectively. Fig 4 confirmed that AI provided most distinguishable summary metrics for activities at every level of activity intensity, while AC and ENMO performed well only for MVPA. In addition, ENMO performed as well as AI for MVPA.

### Comparing MET prediction performance of AI, AC and ENMO

**Fig 5:**
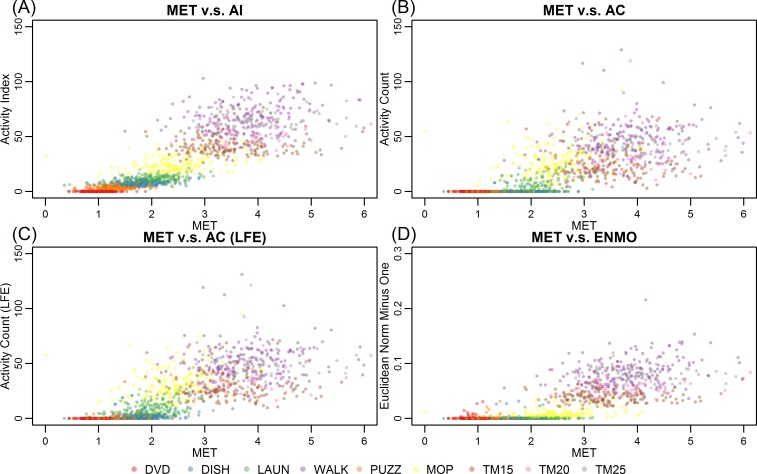
Scatterplots of metabolic equivalents (METs) versus Activity Index (AI) (A), activity count (AC) (B), AC with Low Frequency Extension (LFE) (C) and Euclidean Norm Minus One (ENMO) (D). MET is on *x*-axis for all four plots, while AI, AC, AC (LFE) and ENMO are on the *y*-axis in (A), (B), (C) and (D), respectively. Each point in the figure represents a participant's median METs during a certain activity (rendered in different colors) versus the median AI, AC or ENMO while he/she was performing the same activity.

Fig 5 shows scatterplots of METs versus four metrics: AI, AC, AC (LFE) and ENMO. The METs were positively correlated with all four metrics, with coefficients of determination (*R*^2^) values of 0.72, 0.54, 0.59 and 0.62 for AI, AC, AC (LFE) and ENMO, respectively. Although the ACs and ENMOs were correlated with METs, they were close to 0 for DVD, DISH and PUZZ, while ENMO was close to 0 even for LAUN. In contrast, the MET values for these activities were different, suggesting that ENMO and AC may underperform in terms of predicting low intensity activities. The AC (LFE) exhibited slightly improved sensitivity to sedentary and light activities. In contrast, AI tracked the increase in METs much closer for all activities.

**Fig 6:**
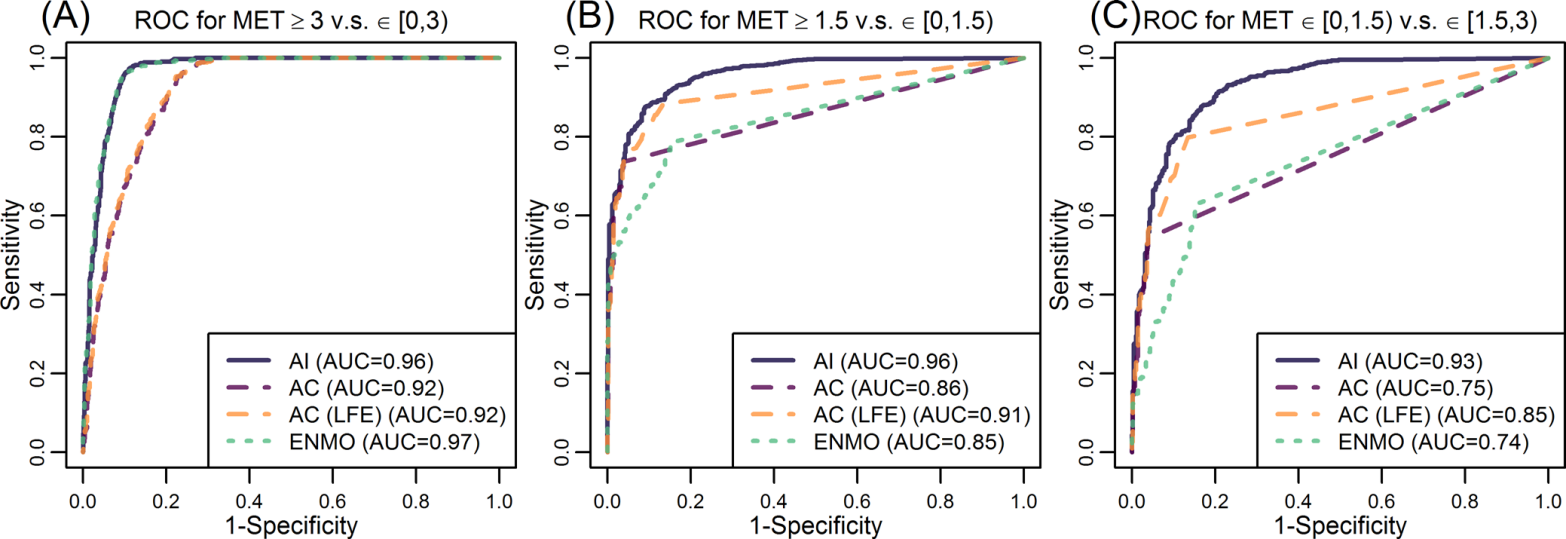
The “receiver operating characteristic” (ROC) curves of Activity Index (AI), activity count (AC), AC with Low Frequency Extension (LFE) and Euclidean Norm Minus One (ENMO) to predict whether metabolic equivalents (MET) is smaller or greater than 3 (A) and 1.5 (B), and whether MET is bigger than 1.5 but smaller than 3 (C). The ROC curves for AI, AC and ENMO are solid, dashed and dotted, respectively, while AC with and without LFE are rendered in purple and orange. The corresponding area under the curve (AUC) of each ROC curve is given in the legend section.

ROC analyses were conducted to further quantify these findings. Fig 6 provides the ROC curves of AI, AC (with and without LFE) and ENMO to classify activity intensity categories such as sedentary (<1.5 METs), light (1.5−3 METs) and MVPA (>3 METs). AI performed better than AC for all activities, while ENMO had slightly worse performance than AC for sedentary and light activities (Fig 6B and 6C, with AUC 0.85 v.s. 0.86 and 0.74 v.s. 0.75). ENMO performed very well when differentiating MVPA and other activities (Fig 6C), with a AUC comparable to that of AI (0.97 v.s. 0.96). The predictive performance of both versions of AC and ENMO was better for MVPA versus light activities (Fig 6A) than light versus sedentary activities (Fig 6C). This indicates that both AC and ENMO are severely limited as classifiers of sedentary and light activities. The AC (LFE) performed better than AC for distinguishing between sedentary and light activities (AUC increased from 0.75 to 0.85 in Fig 6C). The AUC for predicting light versus MVPA was about the same for AC with and without LFE (both 0.92 in Fig 6A). This indicated that LFE does not substantially improve the predictive performance of AC for MVPA.

## Discussion

We proposed AI, a new metric of physical activity based on high-resolution raw accelerometer data. The AI has several desirable properties including transparency, ease of deployment, additivity, and rotational invariance. The new metric was compared to the established AC (with and without LFE) and ENMO using laboratory data from 194 women 60−91 years of age in the OPACH Study. We found that the AI was the best in distinguishing among various types of physical activities across different intensity levels. AI had the best overall performance in terms of predicting energy expenditure expressed in METs, and had better predictive performance for classifying an epoch into various physical activity intensity categories.

As the systematic noise ${\bar{\sigma }}_{i}$ for AI computation is determined using a pre-annotated “non-wear period”, AI could be implemented in large epidemiology studies with low effort. The non-wear periods may be obtained in various ways. First, participants could report non-wear periods. Second, data could be collected while accelerometers are placed on a desk, before being used in the study. In this case, the standard deviations, ${\bar{\sigma }}_{i}$, are accelerometer-specific, instead of participant-specific. Therefore, only one ${\bar{\sigma }}_{i}$ needs to be computed for each accelerometer used in the study, and the same ${\bar{\sigma }}_{i}$ can be used for all participants who use the same accelerometer. Third, a published algorithm [35,36] could be used to identify non-wear periods. Moreover, as in our data analysis, we could further combine all the standard deviations, ${\bar{\sigma }}_{i}$, into a “study-specific” standard deviation, $\bar{\sigma }$, and use this single parameter throughout a study. As long as ${\bar{\sigma }}_{i}$'s are not very different across accelerometers, this approach is reasonable. A simple histogram of ${\bar{\sigma }}_{i}$ can indicate whether the assumption is valid in the study and could identify miss-calibrated accelerometers.

The additivity and rotational invariance are both desired measurement properties that define the new AI as a proper physical activity measure. AI is additive in the sense that AI values in different epochs can be added to provide an aggregated AI that is consistent across resolutions. Additivity is an important self-consistency feature, as it ensures that AI is comparable and generalizable across studies. For instance, if Study A suggests that people use 30 AI per second as the cut-off for light intensity and moderate intensity activities, it is equivalent to suggest 30 × 60 = 1800 AI per minute as the cut-off in Study B that calculates AI per minute. Moreover, AI’s rotational invariance guarantees it remains unchanged while the participant is performing the same type of physical activity with a rotated accelerometer placed at the same location on the body. This property is crucial in practice. In many studies that collect free-living data the device can rotate or tilt when they are being equipped or during the data collection. Our proposed AI theoretically guarantees rotational invariance and reduces noise and bias due to rotation in practical applications. We expect that this will translate in better robustness to rotations and small changes in location on the body.

Although summary measures like AI, AC, and ENMO do not retain all the information in the raw data, analyses based on these metrics should remain a major part of research, due to the substantially reduced data size and explicit interpretation. Fig 1 indicates that the majority of analysis pathways rely on summary measures. Our proposed AI is an open-sources alternative to the popular AC for summarizing the raw data, which is a crucial bridge between the raw data and summary measures. To demonstrate AI is indeed a better method than others to summarize raw data, we showed the AI yielded to more distinct values than AC and ENMO based on the raw data of different activities. AI was also more highly correlated with METs than AC and ENMO, and performed much better while used to classify activities of different intensity categories. Both Evenson et al. [19] and our study showed that although ActiGraph attempted to improve AC using LFE to better capture low-amplitude movement [37], the improvements over standard AC are modest. ENMO is another important open-source summary metric for raw accelerometry data, but was proved to be outperformed by AI for sedentary and light activities. We contend that replacing or complementing AC with AI would provide much-needed transparency for raw data processing and will greatly enhance the characterization of sedentary and light activity.

While the comparison of AI with other metrics was conducted using data from a group of older women, AI’s advantage over AC and ENMO is not limited to this population. Indeed, the approach summarizes information contained in the acceleration time series data, which are independent of population characteristics. While we have demonstrated that AI outperforms AC and ENMO in terms of quantifying sedentary and light activities, it also performs better (than AC) or equally well (as ENMO) for MVPA. Therefore, while populations of other ages also perform activities in these four intensity categories and produce similar acceleration time series, we expect that, in those cases, AI will still have better performance at least for sedentary and light activities. Future studies on youth and young adults, employing a range of physical activity intensities, can explicitly test this.

Our work has several potential limitations. For example, only one type of accelerometer, the ActiGraph GT3X+, was considered in this study. It remains an open problem to compare the AI collected from other devices, though we expect consistent results for well-calibrated accelerometers. Shaker studies or studies using several devices simultaneously could be conducted to answer this type of question. Another limitation is that we only investigated data from hip-worn accelerometers. As many current studies have moved towards wrist-worn accelerometers to improve compliance, it is important to understand how AI performs for wrist-worn accelerometers. A final noteworthy limitation is our focus on women age 60 years and older. Exploration in other samples is warranted. Nevertheless, the proposed AI provides a novel and transparent way to summarize densely sampled raw accelerometry data, and may serve as an alternative to AC.

## Supporting Information

S1 File An activity index for raw accelerometry data and its comparison with other activity metrics: Supplementary Materials. This supplementary material provides technical details and proof of the definition and properties of Activity Index.
(PDF)

## References

[1] Butte NF, Wong WW, Lee JS, Adolph AL, Puyau MR, Zakeri IF. Prediction of energy expenditure and physical activity in preschoolers. Med Sci Sport Exerc. 2014;46: 1216–1226. 10.1249/MSS.0000000000000209 PMC4010568 24195866

[2] John D, Freedson P. ActiGraph and actical physical activity monitors: A peek under the hood. Med Sci Sports Exerc. 2012;44: S86–S89. 10.1249/MSS.0b013e3182399f5e 22157779 PMC3248573

[3] Yang CC, Hsu YL. A review of accelerometry-based wearable motion detectors for physical activity monitoring. Sensors. 2010;10: 7772–7788. 10.3390/s100807772 22163626 PMC3231187

[4] del Rosario M, Redmond S, Lovell N. Tracking the Evolution of Smartphone Sensing for Monitoring Human Movement. Sensors. 2015;15: 18901–18933. 10.3390/s150818901 26263998 PMC4570352

[5] Pedisic Z, Bauman A. Accelerometer-based measures in physical activity surveillance: current practices and issues. Br J Sports Med. 2015;49: 219–223. 10.1136/bjsports-2013-093407 25370153

[6] Bai Y, Welk GJ, Nam YH, Lee JA, Lee JM, Kim Y, et al Comparison of consumer and research monitors under semistructured settings. Med Sci Sports Exerc. 2016;48: 151–158. 10.1249/MSS.0000000000000727 26154336

[7] ActiGraph. What are counts? [Internet]. 2011 [cited 1 Oct 2015]. Available: https://help.theactigraph.com/entries/20723176-What-are-counts-

[8] Bankoski A, Harris TB, McClain JJ, Brychta RJ, Caserotti P, Chen KY, et al Sedentary activity associated with metabolic syndrome independent of physical activity. Diabetes Care. Am Diabetes Assoc; 2011;34: 497–503. 10.2337/dc10-0987 21270206 PMC3024375

[9] Colley RC, Garriguet D, Janssen I, Craig CL, Clarke J, Tremblay MS. Physical activity of Canadian adults: accelerometer results from the 2007 to 2009 Canadian Health Measures Survey. Heal Reports. Statistics Canada Ottawa; 2011;22: 15–23. 21510585

[10] Schrack JA, Zipunnikov V, Goldsmith J, Bai J, Simonsick EM, Crainiceanu C, et al Assessing the “Physical Cliff’': Detailed quantification of age-related differences in daily patterns of physical activity. Journals Gerontol Ser A Biol Sci Med Sci. Oxford University Press; 2014;69: 973–979. 10.1093/gerona/glt199 PMC4095926 24336819

[11] Schrack JA, Zipunnikov V, Goldsmith J, Bandeen-Roche K, Crainiceanu CM, Ferrucci L. Estimating energy expenditure from heart rate in older adults: A case for calibration. PLoS One. Public Library of Science; 2014;9: e93520 10.1371/journal.pone.0093520 24787146 PMC4005766

[12] Troiano RP, Berrigan D, Dodd KW, Mâsse LC, Tilert T, McDowell M. Physical activity in the United States measured by accelerometer. Med Sci Sport Exerc. WILLIAMS & WILKINS; 2008;40: 181–188. 10.1249/mss.0b013e31815a51b3 18091006

[13] Trost SG, Pate RR, Sallis JF, Freedson PS, Taylor WC, Dowda M, et al Age and gender differences in objectively measured physical activity in youth. Med Sci Sport Exerc. Citeseer; 2002;34: 350–355. 10.1097/00005768-200202000-00025 11828247

[14] Welk GJ. Principles of design and analyses for the calibration of accelerometry-based activity monitors. Med Sci Sports Exerc. 2005;37: 501–511. 10.1249/01.mss.0000185660.38335.de 16294113

[15] Freedson PS, Melanson E, Sirard J. Calibration of the Computer Science and Applications, Inc. accelerometer. Med Sci Sport Exerc. 1998;30: 777–781. 10.1097/00005768-199805000-00021 9588623

[16] Puyau MR, Adolph AL, Vohra FA, Butte NF. Validation and calibration of physical activity monitors in children. Obes Res. Wiley Online Library; 2002;10: 150–157. 11886937 10.1038/oby.2002.24

[17] Freedson PS, Pober DM, Janz KF. Calibration of accelerometer output for children. Med Sci Sport Exerc. 2005;37: S523–S530. 10.1249/01.mss.0000185658.28284.ba 16294115

[18] Sasaki JE, John D, Freedson PS. Validation and comparison of ActiGraph activity monitors. J Sci Med Sport. Elsevier; 2011;14: 411–416. 10.1016/j.jsams.2011.04.003 21616714

[19] Evenson KR, Wen F, Herring AH, Di C, LaMonte MJ, Tinker LF, et al Calibrating physical activity intensity for hip-worn accelerometry in women age 60 to 91years: The Women’s Health Initiative OPACH Calibration Study. Prev Med Reports. 2015;2: 750–756. 10.1016/j.pmedr.2015.08.021 PMC4625400 26527313

[20] Crouter SE, Flynn JI, Bassett DR. Estimating physical activity in youth using a wrist accelerometer. Med Sci Sports Exerc. 2015;47: 944–951. 10.1249/MSS.0000000000000502 25207928 PMC4362848

[21] Staudenmayer J, Pober D, Crouter S, Bassett D, Freedson P. An artificial neural network to estimate physical activity energy expenditure and identify physical activity type from an accelerometer. J Appl Physiol. Am Physiological Soc; 2009;107: 1300–1307. 10.1152/japplphysiol.00465.2009 19644028 PMC2763835

[22] Bai J, Goldsmith J, Caffo B, Glass TA, Crainiceanu CM. Movelets: A dictionary of movement. Electron J Stat. Institute of Mathematical Statistics; 2012;6: 559–578. 10.1214/12-EJS684 PMC3535448 23293708

[23] Bao L, Intille SS. Activity recognition from user-annotated acceleration data. Proceedings of the 2nd International Conference on Pervasive Computing. Springer; 2004. pp. 1–17.

[24] He B, Bai J, Zipunnikov V V., Koster A, Caserotti P, Lange-Maia B, et al Predicting human movement with multiple accelerometers using movelets. Med Sci Sports Exerc. LWW; 2014;46: 1859–1866. 10.1249/MSS.0000000000000285 25134005 PMC4137461

[25] Ravi N, Dandekar N, Mysore P, Littman ML. Activity recognition from accelerometer data. Proceedings of the Seventeenth Conference on Innovative Applications of Artificial Intelligence. 2005. pp. 1541–1546.

[26] Staudenmayer J, He S, Hickey A, Sasaki J, Freedson P. Methods to estimate aspects of physical activity and sedentary behavior from high-frequency wrist accelerometer measurements. J Appl Physiol. 2015;119: 396–403. 10.1152/japplphysiol.00026.2015 26112238 PMC4538283

[27] Ellis K, Kerr J, Godbole S, Lanckriet G, Wing D, Marshall S. A random forest classifier for the prediction of energy expenditure and type of physical activity from wrist and hip accelerometers. Physiol Meas. 2014;35: 2191–2203. 10.1088/0967-3334/35/11/2191 25340969 PMC4374571

[28] Bai J, He B, Shou H, Zipunnikov V, Glass TA, Crainiceanu CM. Normalization and extraction of interpretable metrics from raw accelerometry data. Biostatistics. 2014;15: 102–116. 10.1093/biostatistics/kxt029 23999141 PMC4072911

[29] van Hees VT, Gorzelniak L, Dean León EC, Eder M, Pias M, Taherian S, et al Separating movement and gravity components in an acceleration signal and implications for the assessment of human daily physical activity. Müller M, editor. PLoS One. 2013;8: e61691 10.1371/journal.pone.0061691 23626718 PMC3634007

[30] Hildebrand M, Van Hees VT, Hansen BH, Ekelund U. Age-group comparability of raw accelerometer output from wrist- and hip-worn monitors. Med Sci Sport Exerc. 2014;46: 1816–1824. 10.1249/MSS.0000000000000289 24887173

[31] ActiGraph. Idle Sleep Mode Explained [Internet]. 2012 [cited 1 Oct 2015]. Available: https://help.theactigraph.com/entries/21625711-Idle-Sleep-Mode-Explained

[32] ActiGraph. Low Frequency Extension Explained [Internet]. 2012 [cited 1 Oct 2015]. Available: https://help.theactigraph.com/entries/21767838-Low-Frequency-Extension-Explained

[33] Cain KL, Conway TL, Adams MA, Husak LE, Sallis JF. Comparison of older and newer generations of ActiGraph accelerometers with the normal filter and the low frequency extension. Int J Behav Nutr Phys Act. 2013;10: 51 10.1186/1479-5868-10-51 23618461 PMC3641979

[34] Borg G, Linderholm H. Perceived exertion and pulse rate during graded exercise in various age groups. Acta Med Scand. 1967;181: 192–206.

[35] Choi L, Liu Z, Matthews CE, Buchowski MS. Validation of accelerometer wear and nonwear time classification algorithm. Med Sci Sport Exerc. NIH Public Access; 2011;43: 357–364. 10.1249/MSS.0b013e3181ed61a3 PMC3184184 20581716

[36] Choi L, Ward SC, Schnelle JF, Buchowski MS. Assessment of Wear/Nonwear Time Classification Algorithms for Triaxial Accelerometer. Med Sci Sport Exerc. 2012;44: 2009–2016. 10.1249/MSS.0b013e318258cb36 PMC3443532 22525772

[37] Feito Y, Garner HR, Bassett DR. Evaluation of ActiGraph’s Low-Frequency Filter in Laboratory and Free-Living Environments. Med Sci Sport Exerc. 2015;47: 211–217. 10.1249/MSS.0000000000000395 24870583

