# Supplementary material for: An Activity Index for Raw Accelerometry Data and Its Comparison with Other Activity Metrics
Source: PLoS One. 2016 Aug 11;11(8):e0160644. doi: 10.1371/journal.pone.0160644 (PMC4981309; doi:10.1371/journal.pone.0160644)
Supplement: S1 File — This supplementary material provides technical details and proof of the definition and properties of Activity Index. (PDF) [file pone.0160644.s001.pdf]

An activity index for raw accelerometry data and its comparison with other activity metrics: Supplementary Materials

Jiawei Bai[1*], Chongzhi Di[2], Luo Xiao[3], Kelly R. Evenson[4], Andrea Z. LaCroix[5], Ciprian M. Crainiceanu[1] and David M. Buchner[6]

[1] Department of Biostatistics, Johns Hopkins University, Baltimore, Maryland, US

[2] Division of Public Health Sciences, Fred Hutchinson Cancer Research Center, Seattle, Washington, US

[3] Department of Statistics, North Carolina State University at Raleigh, Raleigh, North Carolina, US

[4] Department of Epidemiology, Gillings School of Global Public Health, University of North Carolina – Chapel Hill, Chapel Hill, North Carolina, US

[5] Division of Epidemiology, Department of Family Medicine and Public Health, University of California, San Diego, La Jolla, California, US

[6] Department of Kinesiology and Community Health, University of Illinois at Urbana-Champaign, Champaign, Illinois, US

* Corresponding author

Email: jbai@jhsph.edu

We first illustrate in detail how Activity Index (AI) is formalized. Then, we discuss the three properties of AI more rigorously.

Definitions and mathematical formula of AI

We first introduce notations. Denote the data by $\boldsymbol{X}_i(t) = \{X_{i1}(t), X_{i2}(t), X_{i3}(t)\}$ $(t = 1,2,\ldots,T_i)$, where T_i is the length of the accelerometer time series for Participant i. Let f denote the sample rate ($f = 30$Hz in our study) and H be the window size of raw data to be summarized into one AI measure, where $H \geq f$ (windows size is no less than the sample rate). Thus, we limit the smallest window length to calculate AI (i.e., epoch length) to be 1-second. Define $\sigma_i(t;H) = \{\sigma_{i1}(t;H), \sigma_{i2}(t;H), \sigma_{i3}(t;H)\}$ to be the standard deviation function for data from a time interval of length H starting at time t. More specifically,

$$\sigma_{im}(t) = \sqrt{\frac{\sum_{h=0}^{H-1}\{X_{im}(t+h) - \sum_{k=0}^{H-1} X_{im}(t+k)/H\}^2}{H}}, m = 1,2,3. \qquad (S1)$$

As described in the main text, the $\sigma_{im}^2(t;H)$ are axis- and participant-specific moving variance that characterize the variation of acceleration along each axis in the window of length H starting at t. We then introduce $\bar{\sigma}_i$ as the systematic standard deviation when the device is placed steady (not moving). Formally, for every $t/H \in \mathbb{Z}$,

$$\bar{\sigma}_i = \frac{\sum_{t\in\mathcal{T}_i \text{ and } t/H\in\mathbb{Z}} \sqrt{\frac{1}{3}\{\sigma_{i1}^2(t;H) + \sigma_{i2}^2(t;H) + \sigma_{i3}^2(t;H)\}}}{|\{t \in \mathcal{T}_i \; and \; t/H \in \mathbb{Z}\}|}, \qquad (S2)$$

where $\mathcal{T}_i$ stands for collection of time points t during which the device is considered steady. Constraint $t/H \in \mathbb{Z}$ guarantees $\sigma_{im}(t)$'s are only computed at time point which is a multiple of H, so that $|\{t \in \mathcal{T}_i \; and \; t/H \in \mathbb{Z}\}|$ is the number of complete epochs (of length H) in $\mathcal{T}_i$. Usually, $\mathcal{T}_i$ can be specified by the users themselves, and examples include the time period during which

the accelerometer is placed on the table. The variances $\bar{\sigma}_i^2$ are the participant-specific systematic noise of acceleration and are calculated as the variance of the observed raw accelerometry data across all three axes in periods of non-movement.

Formally, the Activity Index $AI_i^{new}(t;H)$ is given by either

$$AI_i^{ABS}(t;H) = \sqrt{\max\left(\frac{1}{3}\left\{\sum_{m=1}^{3} \sigma_{im}^2(t;H) - \bar{\sigma}_i^2\right\}, 0\right)} \qquad (S3)$$

in absolute scale or

$$AI_i^{REL}(t;H) = \sqrt{\max\left(\frac{1}{3}\left\{\sum_{m=1}^{3} \frac{\sigma_{im}^2(t;H) - \bar{\sigma}_i^2}{\bar{\sigma}_i^2}\right\}, 0\right)} \qquad (S4)$$

in relative scale. As $AI_i^{new}(t;H)$ is defined at every $t \in [1, T_i]$, it is guaranteed to exist for each second. Note that Equations (S3) and (S4) are identical to Equations (1) and (2) in the main text, respectively.

Note that the $AI_i^{new}(t;H)$ is related to, but different from the original AI_0 defined by

$$AI_i^0(t;H) = \max\left(\frac{1}{3}\left\{\sum_{m=1}^{3} \frac{\sigma_{im}(t;H) - \bar{\sigma}'_{im}}{\bar{\sigma}'_{im}}\right\}, 0\right), \qquad (S5)$$

where $\bar{\sigma}'_{im}$ is the systematic noise on each axis computed using the inactive time period, which depends on choosing a threshold C for the distribution of AI_0 in all epochs. The main differences between the original and new AIs are how the systematic noise variance is calculated and how signals from three axes are combined. As will be shown below, compared to the original AI_0, the new AI has a few advantages, including ease for implementation especially in large-scale studies and nice mathematical properties such as additivity and rotational invariance.

Properties of AI

Easy implementation

As discussed in Bai et al. (2014), the original AI_0 depends on the choice of a threshold C to determine whether a participant was active or not in each second to calculate the systematic noise $\sigma'_{im}(t;H)$. Although $AI_i^0(t;H)$ was shown not to vary too much with C, this process was tedious and infeasible in large scale studies and might not lead to an AI_0 comparable across different studies. In contrast, Equation (S2) implies that $\bar{\sigma}_i$ is determined using "non-wear periods", which could easily be identified either by existing algorithms, by pilot studies or via participants' self-annotations.

Additivity

Additivity of $AI_i^{new}(t;H)$ could be formalized as follow. First, let $H=f$ and calculate second-by-second $AI_i^{new}(t;f)$. Then, $AI_i^{new}(t;f)$'s are summed up to generate AI in longer epochs. For example, minute-by-minute AI satisfies

$$AI_i^{new}(t;60f)=\sum_{s=0}^{59} AI_i^{new}(t+sf;f),$$

where $AI_i^{new}(t;60f)$ is only defined at $\{t: t/(60f)\in\mathbb{Z}\}$.

Rotational Invariance

In this section we formally prove the rotational invariance of $AI_i^{new}(t;H)$. This property is achieved by i) replacing axis-specific $\bar{\sigma}_{i1}$, $\bar{\sigma}_{i2}$ and $\bar{\sigma}_{i3}$ in (S5) with one single $\bar{\sigma}_i$ as in (S3) or (S4) and ii) change standard deviations to variances. Since $AI_i^{ABS}(t;H)$ and $AI_i^{REL}(t;H)$ are directly proportional, we only give the proof of rotational invariance for $AI_i^{REL}(t;H)$ and one can easily follow the same flow to verify such property for $AI_i^{ABS}(t;H)$. Let an orthogonal 3×3 matrix

$R = \{r_{mm}\}_{3\times3}$ be the rotation matrix. In another word, rotating $\boldsymbol{X}_i(t) = \{X_{i1}(t), X_{i2}(t), X_{i3}(t)\}$ with respect to R could be formally written as

$$\boldsymbol{X}_i^*(t) = \left\{\sum_{m=1}^{3} r_{1m}X_{im}(t)\,, \sum_{m=1}^{3} r_{2m}X_{im}(t)\,, \sum_{m=1}^{3} r_{3m}X_{im}(t)\right\},$$

while the rotated $\boldsymbol{X}_i^*(t) = \{X_{i1}^*(t), X_{i2}^*(t), X_{i3}^*(t)\}$ satisfies

$$[X_{i1}^*(t)]^2 + [X_{i2}^*(t)]^2 + [X_{i3}^*(t)]^2 = [X_{i1}(t)]^2 + [X_{i2}(t)]^2 + [X_{i3}(t)]^2, \qquad (S6)$$

as such rotation does not change the distance from any point to the origin. Further, introduce the mean functions $\mu_{im}(t;N) = \frac{1}{N}\sum_{n=1}^{N} X_{im}(t+n-1)$ and $\mu_{im}^*(t;N) = \frac{1}{N}\sum_{n=1}^{N} X_{im}^*(t+n-1)$ for convenience. It can be verified that the point

$$\{\mu_{i1}(t;N), \mu_{i2}(t;N), \mu_{i3}(t;N)\}$$

is the counterpart of the point

$$\{\mu_{i1}^*(t;N), \mu_{i2}^*(t;N), \mu_{i3}^*(t;N)\}$$

before the rotation R. Since Equation (S6) holds for any pair of original and rotated points, it guarantees

$$[\mu_{i1}^*(t;N)]^2 + [\mu_{i2}^*(t;N)]^2 + [\mu_{i3}^*(t;N)]^2 = [\mu_{i1}(t;N)]^2 + [\mu_{i2}(t;N)]^2 + [\mu_{i3}(t;N)]^2. \qquad (S7)$$

Finally, together with (S1) and (S4), the rotational invariance can be verified as follow

$$AI_i^{REL}(t;H) = \sqrt{\max\left(\frac{1}{3}\left\{\sum_{m=1}^{3} \frac{\sigma_{im}^2(t;H) - \bar{\sigma}_i^2}{\bar{\sigma}_i^2}\right\}, 0\right)}$$

$$= \sqrt{\max\left(\frac{1}{3\bar{\sigma}_i^2}\sum_{m=1}^{3} \sigma_{im}^2(t;H) - 1, 0\right)}$$

$$= \sqrt{\max\left(\frac{1}{3H\bar{\sigma}_i^2}\sum_{m=1}^{3}\sum_{h=0}^{H-1}\{X_{im}(t+h)-\mu_{im}(t;H)\}^2 - 1, 0\right)}$$

$$= \sqrt{\max\left(\frac{1}{3H\bar{\sigma}_i^2}\sum_{h=0}^{H-1}\left[\sum_{m=1}^{3}\{X_{im}(t+h)\}^2 - H\sum_{m=1}^{3}\{\mu_{im}(t;H)\}^2\right] - 1, 0\right)}$$

$$= \sqrt{\max\left(\frac{1}{3H\bar{\sigma}_i^2}\sum_{h=0}^{H-1}\left[\sum_{m=1}^{3}\{X_{im}^*(t+h)\}^2 - H\sum_{m=1}^{3}\{\mu_{im}^*(t;H)\}^2\right] - 1, 0\right)}$$

$$= AI_i^{REL^*}(t;H),$$

where $AI_i^{REL^*}(t;H)$ is the relative scale AI based on rotated data $\boldsymbol{X}_i^*(t) = \{X_{i1}^*(t), X_{i2}^*(t), X_{i3}^*(t)\}$.
